# Effects of Nrf2 Deficiency on Bone Microarchitecture in an Experimental Model of Osteoporosis

**DOI:** 10.1155/2014/726590

**Published:** 2014-07-07

**Authors:** Lidia Ibáñez, María Luisa Ferrándiz, Rita Brines, David Guede, Antonio Cuadrado, Maria José Alcaraz

**Affiliations:** ^1^Department of Pharmacology, University of Valencia, Avenue Vicent A. Estellés s/n, Burjasot, 46100 Valencia, Spain; ^2^Trabeculae S.L., Parque Tecnolóxico de Galicia, San Cibrao das Viñas, 32900 Ourense, Spain; ^3^Centro de Investigación en Red sobre Enfermedades Neurodegenerativas (CIBERNED), Department of Biochemistry and Alberto Sols Biomedical Research Institute UAM-CSIC, Faculty of Medicine, Autonomous University of Madrid, Avenue Arzobispo Morcillo 4, 28029 Madrid, Spain

## Abstract

*Objective*. Redox imbalance contributes to bone fragility. We have evaluated the in vivo role of nuclear factor erythroid derived 2-related factor-2 (Nrf2), an important regulator of cellular responses to oxidative stress, in bone metabolism using a model of postmenopausal osteoporosis. *Methods*. Ovariectomy was performed in both wild-type and mice deficient in Nrf2 (Nrf2^−/−^). Bone microarchitecture was analyzed by *μ*CT. Serum markers of bone metabolism were also measured. Reactive oxygen species production was determined using dihydrorhodamine 123. *Results*. Sham-operated or ovariectomized Nrf2^−/−^ mice exhibit a loss in trabecular bone mineral density in femur, accompanied by a reduction in cortical area in vertebrae. Nrf2 deficiency tended to increase osteoblastic markers and significantly enhanced osteoclastic markers in sham-operated animals indicating an increased bone turnover with a main effect on bone resorption. We have also shown an increased production of oxidative stress in bone marrow-derived cells from sham-operated or ovariectomized Nrf2^−/−^ mice and a higher responsiveness of bone marrow-derived cells to osteoclastogenic stimuli in vitro. *Conclusion*. We have demonstrated in vivo a key role of Nrf2 in the maintenance of bone microarchitecture.

## 1. Introduction

Multiple pathogenetic mechanisms are responsible for loss of bone mass and skeletal microarchitectural deterioration leading to osteoporosis which represents a major challenge to our health systems. Excessive bone resorption or an inadequate bone formation in response to increased resorption during bone remodeling results in skeletal fragility [1]. Epidemiological studies have revealed that the majority of the affected individuals are postmenopausal women. It is estimated that in developed countries around 50 percent of women aged 50 and older will sustain an osteoporotic fracture during their lifetime. Osteoporotic fractures are associated with chronic pain and greatly impact the quality of life. Experimental findings support the concept that estrogen deficiency leads to increased bone turnover and plays a key role in osteoporosis [2].

Recent studies have demonstrated an important contribution of redox imbalance to bone fragility [3] and biochemical markers of oxidative stress are significantly increased in osteoporosis [4, 5]. In addition, menopause-related estrogen withdrawal might contribute to make bone more vulnerable to oxidative injury. Loss of estrogens decreases defensive mechanisms against oxidative stress and accelerates the effects of aging on bone [6]. Therefore, reduced antioxidant levels would enhance bone resorption [7] whereas the reduction in oxidative stress may provide protection against osteoporosis in aged and ovariectomized rats [8].

Nuclear factor erythroid derived 2-related factor-2 (Nrf2) plays an important role in the cellular defense against oxidative stress by induction of antioxidative and phase-2 detoxifying enzymes. This transcription factor regulates the expression of bone marrow stromal antioxidants and phase 2 enzymes as well as the susceptibility of these cells to oxidative and electrophilic stress [9]. In addition, Nrf2 downregulates inflammatory processes [10–12] and innate immune responses [13, 14]. Loss of Nrf2 leads to an increased susceptibility to radiation-induced bone loss [15] and recent in vitro studies have shown a role for this transcription factor in receptor activator of nuclear factor *κ*B ligand (RANKL) induced osteoclastogenesis through modulation of reactive oxygen species (ROS) signaling [16, 17]. In contrast, some reports have suggested a negative effect of Nrf2 on bone mineralization [18, 19]. However, the influence of Nrf2 deficiency on bone metabolism and microstructure in vivo and the possible role of Nrf2 in osteoporosis are not completely understood.

Ovariectomized mice may serve as an appropriate model for type II osteoporosis [20]. Approaches coupling in vitro observations with events in vivo allow a better understanding of complex biological interactions and disease mechanisms. The present study was designed to evaluate the in vivo effects of Nrf2 deficiency in a model of postmenopausal osteoporosis.

## 2. Materials and Methods

### 2.1. Animals and Study Design

All studies were performed in accordance with European Union regulations for the handling and use of laboratory animals. The protocols were approved by the institutional Animal Care and Use Committee (Comité de Etica en Experimentación Animal, University of Valencia and Autonomous University of Madrid, Spain). The C57BL/6J Nrf2 knockout mice were kindly provided by Dr. Masayuki Yamamoto (University of Tsukuba, Japan). Genotyping of wild-type (Nrf2^+/+^) C57BL/6J mice and Nrf2-deficient (Nrf2^−/−^) littermates was done as reported previously [22]. Female Nrf2^−/−^ and wild-type C57BL/6J mice between 6 and 9 months of age were included in this study. Animals of each strain were randomly allocated to ovariectomy (OVX) (*n* = 20) or Sham groups (*n* = 20). Surgery was performed after the mice were anesthetized with isoflurane and treated with butorfanol (2 mg/kg, s.c.). Ovaries of the mice in OVX group were removed through a midline incision of the skin and flank incisions of the peritoneum. The skin incision was then closed with metallic clips. Mice in the sham-operated group were anesthetized and sutured without removal of the ovaries. All mice were maintained in cages with a 12-hour light/dark cycle and free access to standard diet and water. Mice were housed and cared for by the veterinary staff in accredited facilities and were routinely screened for health status. On day 30 after surgery, mice were anesthetized, blood samples were taken by retro-orbital puncture, and animals were killed by cervical dislocation. Hind paws and L3–L5 vertebrae from randomly selected animals in each group were isolated and frozen for *μ*CT and histomorphometric analysis.

### 2.2. Histomorphometric Analysis

Lumbar spines were dissected (L3–L5) and fixed in 10% formalin. After decalcification in 10% EDTA, the specimens were processed for paraffin embedding. Tissue sections (7 *μ*m) were stained with hematoxylin and eosin to measure trabecular and cortical bone area, and osteoblast number relative to bone perimeter (N.Ob/BPm, mm^−1^) or with TRAP and counterstained with hematoxylin and aniline blue to determine the percentage of bone surface covered by osteoclasts (Oc.S/BS, %).

### 2.3. Measurement of Bone Markers

Enzyme-linked immunoassay (ELISA) kits were used to determine serum levels of tartrate-resistant acid phosphatase (TRAP)-5b, with sensitivity of 0.1 U/L, and C-terminal telopeptides of type I collagen (CTX-I), with sensitivity of 2.0 ng/mL (IDS, Paris, France). Serum levels of osteocalcin, osteoprotegerin (OPG), and RANKL were determined by Luminex, with sensitivity of 7.0, 2.3, and 3.3 pg/mL, respectively (Millipore Corporation, Billerica, MA, USA). Alkaline phosphatase (ALP) levels in serum were determined as previously described [21].

### 2.4. X-Ray Microcomputed Tomography (*μ*CT)

Hind paws were kept wrapped in gauzes soaked in 0.9% NaCl at −20°C until their analysis by *μ*CT. Samples were analyzed with a SkyScan 1172 *μ*CT equipment (Bruker microCT NV, Kontich, Belgium). Three-dimensional trabecular microarchitecture was analyzed at the distal metaphysis of the femur in a region of 1.5 mm. Samples were imaged with an X-ray tube voltage of 50 kV, current of 200 *μ*A, at a scanning voxel size of 5.5 *μ*m, and with the use of an aluminum filter (0.5 mm in thickness). The scanning total angular rotation was 185° with an angular increment of 0.4°. Datasets were reconstructed using a modified Feldkamp algorithm [23] and segmented into binary images using adaptive local thresholding previous to analysis.

### 2.5. Isolation of Bone Marrow Cells

Bone marrows were flushed out of femur and tibia with DMEM (Sigma-Aldrich, St Louis, MO, USA) containing penicillin (100 U/mL) and streptomycin (100 mg/mL), supplemented with 10% fetal bovine serum (Sigma-Aldrich) (complete DMEM) using a 25-gauge needle. Cells were treated with red blood cells lysis buffer (0.15 M NH_4_Cl, 1 mM KHCO_3_, 0.1 mM Na_2_EDTA, pH 7.4), washed twice with complete DMEM, and seeded in 96-well tissue culture plates at 5 × 10^5^ cells per well (oxidative stress quantification) or 3 × 10^5^ cells per well (osteoclast differentiation).

### 2.6. Oxidative Stress Quantification

ROS production in bone marrow-derived cells was determined using dihydrorhodamine 123 (Molecular Probes, Invitrogen S.A., Barcelona, Spain), which is oxidized to fluorescent rhodamine 123 (485 nm (excitation)/534 nm (emission)). Cells were washed with DMEM (Sigma-Aldrich), incubated with dihydrorhodamine 123 (5 *μ*M) in DMEM for 10 min at 37°C and then 12-O-tetradecanoylphorbol-13 acetate (TPA, 200 ng/mL) was added and cells were incubated for 30 min at 37°C. Cells were washed, resuspended in PBS, and analyzed by flow cytometry (FACSCanto, Becton Dickinson, Franklin Lake, NJ, USA). Each experiment was done in triplicate.

### 2.7. Osteoclast Differentiation

Bone marrow-derived cells were cultured in complete DMEM medium containing macrophage-colony stimulating factor (M-CSF) (30 ng/mL) and RANKL (50 ng/mL). Medium was changed every 2 days. TRAP staining was performed after 8 days of culture. Cells were fixed with 4% paraformaldehyde in PBS for 5 min, washed, and incubated in 0.2 M acetate buffer (0.2 M sodium acetate, 0.05 M tartaric acid, and water; pH 5.0) for 20 min at 37°C. Cells were then washed and incubated in a solution containing distilled water, 0.5 mg/mL naphthol AS-MX phosphate (Sigma-Aldrich), and 1.1 mg/mL fast red violet LB salt (Sigma-Aldrich) for 3 h at 37°C. Finally, cells were washed and counterstained with hematoxylin/aniline blue, and TRAP+ multinucleated cells were counted.

### 2.8. Statistical Analysis

The results are presented as mean ± SD. The level of statistical significance was determined by one-way ANOVA followed by Bonferroni's post test.

## 3. Results

### 3.1. Effects on Bone Architecture in Femur

**Figure 1:**
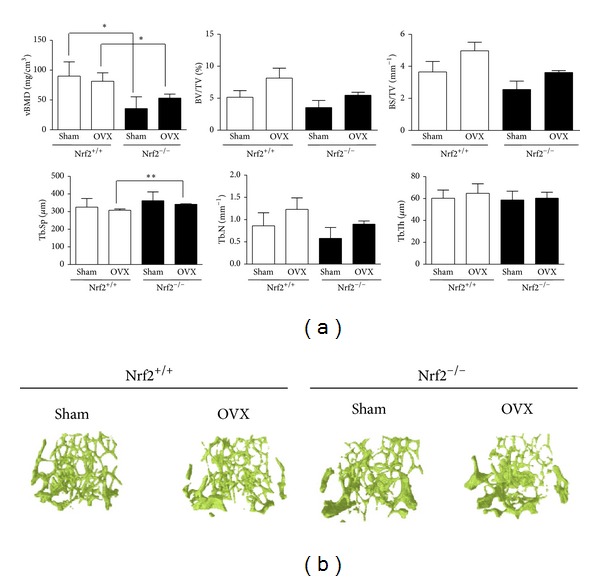
Structure of trabecular bone of femur. (a) Three-dimensional *μ*CT analysis. vBMD: volumetric bone mineral density; BV/TV: bone volume fraction; BS/TV: bone surface density; Tb.Sp: trabecular spacing; Tb.N: trabecular number; Tb.Th: trabecular thickness; OVX: ovariectomy. Results are expressed as mean ± SD (*n* = 3). *P < 0.05, **P < 0.01. (b) Representative *μ*CT images of trabecular bone of femur are shown.

The *μ*CT analysis indicated that Nrf2 deficiency in either sham-operated or ovariectomized mice affected bone architecture in femur. As shown in Figures 1(a) and 1(b), Nrf2 deficiency significantly reduced volumetric bone mineral density (vBMD) in trabecular bone and a tendency was observed towards a reduction in bone volume fraction (BV/TV) and bone surface density (BS/TV). In addition, Nrf2 deficient mice also tended to show lower values of trabecular number (Tb.N), accompanied with a higher trabecular spacing (Tb.Sp) in ovariectomized mice.

### 3.2. Effects on Vertebral Bone

**Figure 2:**
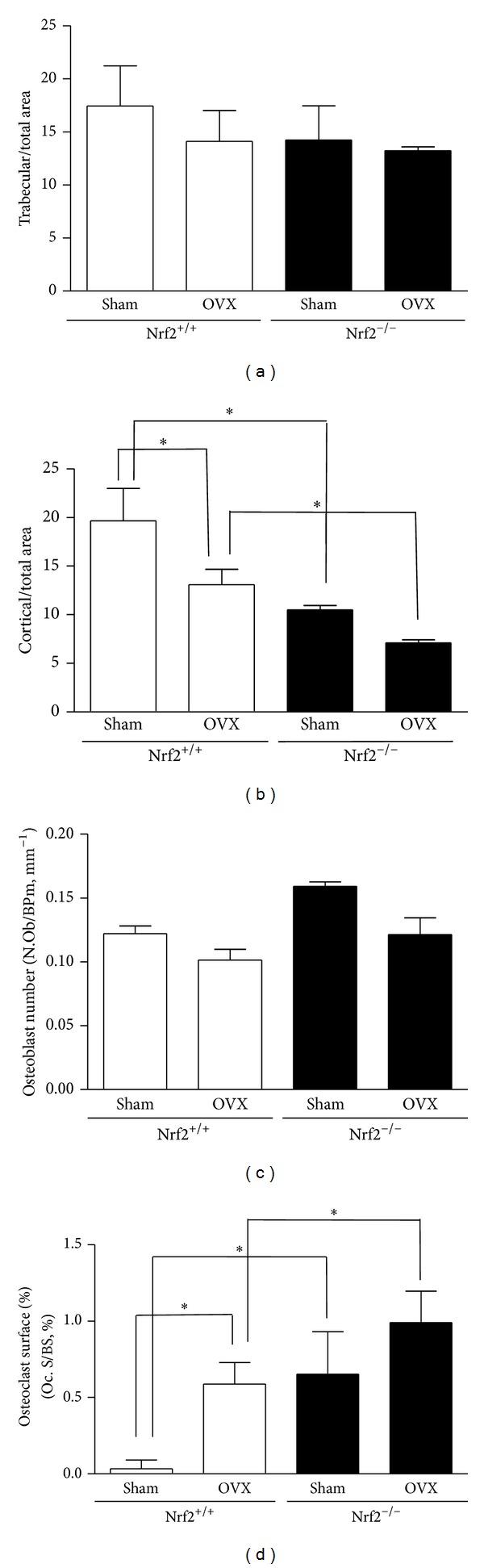
Bone histomorphometric analysis in lumbar vertebrae. The relative surface of trabecular bone (trabecular/total area) and cortical bone (cortical/total area) as well as the osteoblast number relative to bone perimeter (N.Ob/BPm, mm^−1^) and the percentage of bone surface covered by osteoclasts (Oc.S/BS, %) were measured. OVX: ovariectomy. Data represent mean ± SD (*n* = 8). *P < 0.05.

Histomorphometric analysis of vertebral bone showed a significant reduction in the ratio cortical area/total area accompanied with a tendency to a lower trabecular area/total area ratio in sham-operated or ovariectomized Nrf2-deficient mice compared with wild-type controls (Figure 2). In addition, a tendency to higher osteoblast numbers was observed in the presence of Nrf2 deficiency in either sham-operated or ovariectomized animals. Interestingly, osteoclast surface was significantly higher in Nrf2^−/−^ mice relative to their corresponding wild-type controls. Ovariectomy tended to reduce osteoblast numbers and significantly increased osteoclast surface.

### 3.3. Effects on Serum Markers of Bone Turnover

**Figure 3:**
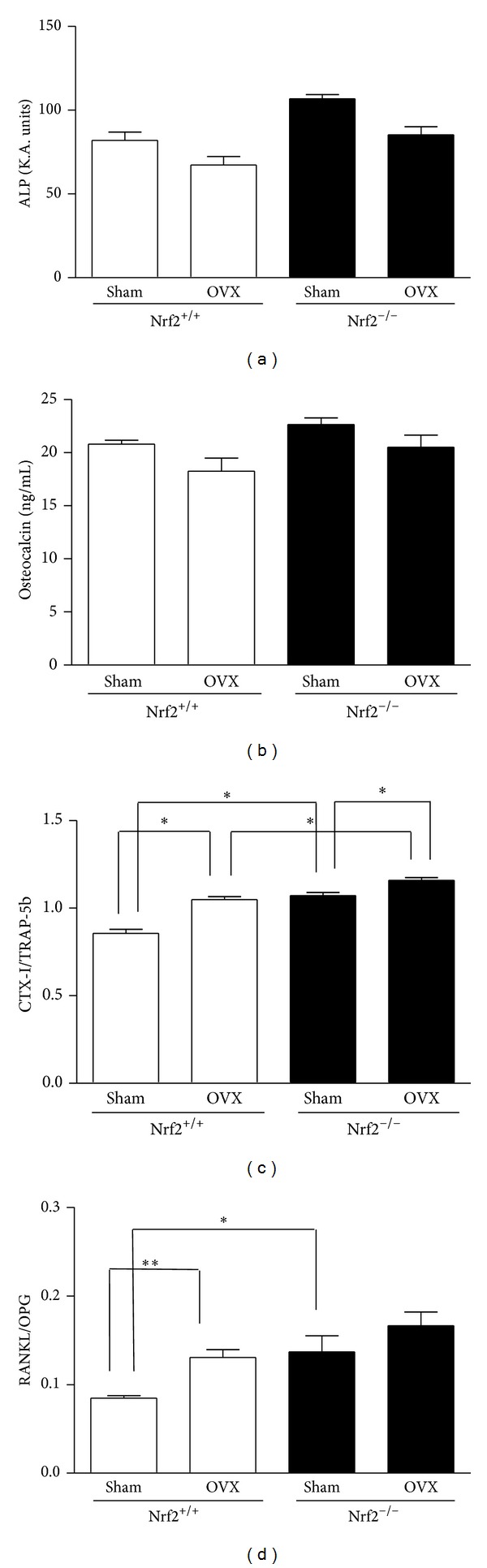
Serum levels of bone turnover markers. ALP was assessed by the method of Kind and King [21]. TRAP-5b and CTX-I levels were measured by ELISA. Osteocalcin, OPG, and RANKL levels were measured by Luminex. OVX: ovariectomy. Results are expressed as mean ± SD (*n* = 8). *P < 0.05, **P < 0.01.

No significant effects were obtained for serum ALP and osteocalcin (early and late markers of osteoblast differentiation, resp.) levels (Figure 3) although a tendency to higher values was observed in the presence of Nrf2 deficiency. CTX-I and TRAP-5b levels indicate osteoclast activity and osteoclast number, respectively, and the ratio CTX-I/TRAP-5b is a sensitive resorption index [24]. CTX-I/TRAP-5b as well as the ratio RANKL/OPG, an index of osteoclastic activation, increased in Nrf2^−/−^ mice. In addition, ovariectomy augmented CTX-I/TRAP-5b and RANKL/OPG.

### 3.4. Osteoclast Differentiation

**Figure 4:**
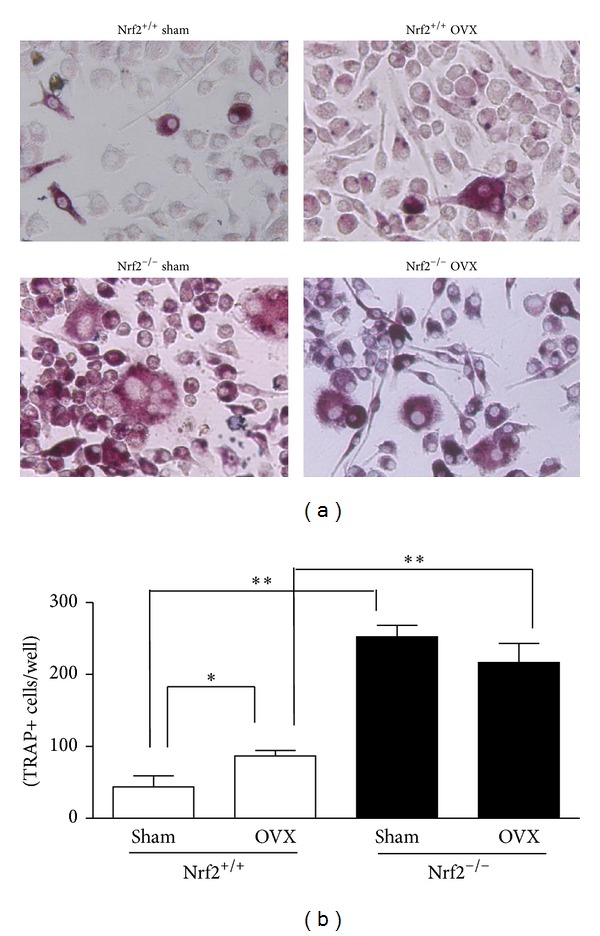
Osteoclast differentiation of bone marrow-derived cells. Cells were cultured in complete DMEM medium containing M-CSF (30 ng/mL) and RANKL (50 ng/mL). TRAP staining was performed after 8 days of culture and TRAP+ multinucleated cells were counted. (a) Representative images, original magnification ×400. OVX: ovariectomy. (b) Cell quantification. Data represent mean ± SD (*n* = 4). *P < 0.05, **P < 0.01.

Increased serum levels of TRAP-5b/CTX-I and RANKL/OPG as well as osteoclast surface in vertebrae sections were observed in Nrf2^−/−^ mice. In order to confirm the response to relevant osteoclastogenic stimuli [16, 17] in our experimental model, we used bone marrow-derived cells of mice from the different experimental groups treated with M-CSF and RANKL.  Figure 4 shows that deficiency in Nrf2 enhanced in vitro osteoclast differentiation in either sham-operated or ovariectomized animals.

### 3.5. Oxidative Stress

**Figure 5:**
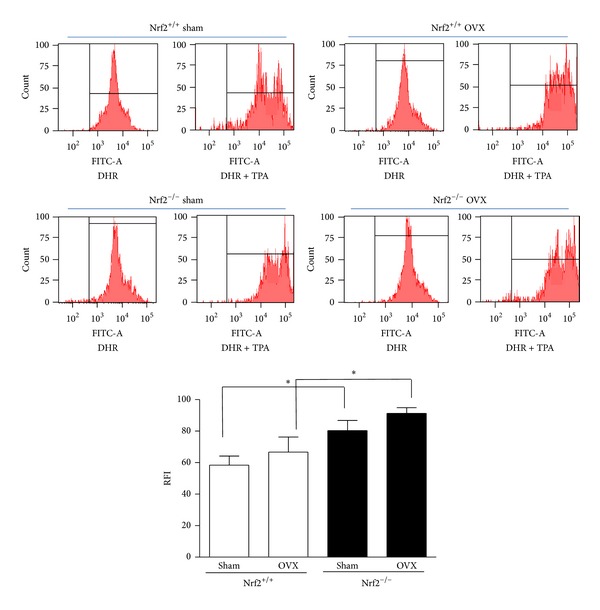
Production of oxidative stress by bone marrow-derived cells. Oxidative stress was measured in bone marrow-derived cells stimulated with TPA by flow cytometry using dihydrorhodamine 123 (DHR). RFI: relative fluorescence intensity; OVX: ovariectomy. Data represent mean ± SD (*n* = 4). *P < 0.05.

The possible effects on ROS production were investigated in our experimental model. For this purpose, we assessed oxidative stress in bone marrow-derived cells by quantifying intracellular ROS accumulation. As expected, Nrf2 deficiency resulted in a higher oxidative stress either in sham-operated or ovariectomized mice (Figure 5). In addition, ovariectomy tended to increase ROS production.

## 4. Discussion

A wide range of evidence indicates that Nrf2 regulates the expression of genes involved in cellular detoxification and maintains cellular redox homeostasis [25, 26]. In this work, we focused on the possible role of Nrf2 in bone metabolism because oxidative stress is an important mediator of bone loss in estrogen deficiency. Analysis of bone parameters in Nrf2^−/−^ mice and their wild-type littermates either in the absence or presence of ovariectomy showed that Nrf2 is required for the maintenance of bone quality. We demonstrate that mice deficient in Nrf2 exhibit a loss in trabecular bone mineral density in femora and a reduction in cortical bone area in vertebrae. These changes induced by Nrf2 deficiency may result in bone fragility and fracture risk. Nevertheless, enhanced bone formation activity has been reported in 9-week-old Nrf2^−/−^ mice [27]. Further studies are needed to determine the influence of age on Nrf2 regulatory effects in bone.

This study has demonstrated an increased production of ROS by bone marrow cells from either sham-operated or ovariectomized Nrf2-deficient mice which is consistent with the constitutive lower levels of antioxidants reported in cells from these animals [9]. A wide range of evidence indicates that ROS are intermediaries in osteoclast metabolism leading to osteoclast differentiation or activation [7, 28]. Therefore, ROS are mediators of RANKL signaling in osteoclasts [29] although they do not seem to be directly involved in the bone resorption process [30]. We have shown that Nrf2 deficiency increases osteoclast numbers in vivo which would be the consequence of a higher osteoclastogenic stimulation. Previous in vitro studies have shown higher levels of osteoclast differentiation induced by RANKL and bone resorption using cells from Nrf2-deficient mice [17, 27]. In addition to a higher responsiveness of bone marrow-derived cells to osteoclastogenic stimuli in vitro, we have demonstrated the consequences of Nrf2 deficiency on relevant in vivo biomarkers such as the ratio RANKL/OPG in serum which was significantly increased leading to enhanced osteoclast differentiation in vivo. Serum levels of resorption markers and the *μ*CT and histomorphometric analyses confirmed that the increased osteoclast number reflects an increased bone resorption in vivo. We have also shown a high production of oxidative stress in bone marrow-derived cells after ovariectomy. The accumulation of ROS in these cells would activate T cells through CD80 upregulation on dendritic cells [31]. In addition, the lowering of antioxidants by estrogen deficiency may sensitize osteoclasts to osteoclastogenic signals promoting bone resorption [7]. Nevertheless, our results in this experimental model suggest that the maximal osteoclastogenic response to oxidative stress would be elicited by Nrf2 deficiency as ovariectomy did not further increase it.

On the other hand, an increased oxidative stress may contribute to the inhibition of osteoblast differentiation [32] and proliferation [33] or the induction of cell death [34]. Previous studies have suggested the involvement of Nrf2 in bone mineralization. Stable overexpression of Nrf2 inhibits runt-related transcription factor 2-dependent osteocalcin promoter activity and the formation of mineralized matrix in MC3T3-E1 cells [18]. Recent data also support a negative role for Nrf2 in osteoblast differentiation and mineralization [27]. In contrast, the lack of Nrf2 may facilitate the negative effects of ionizing radiation on osteoblast mineralization [15]. Our data indicate that Nrf2 deficiency tended to increase the in vivo levels of osteoblastic markers and significantly enhanced osteoclastic markers in sham-operated animals. Therefore, we have shown that Nrf2 deficiency leads to an enhanced bone turnover and the predominance of bone resorption. The results of our study thus support a role for Nrf2 in bone metabolism in vivo.

Development of therapies for improving bone formation and bone repair has considerable impact. We found that genetic deletion of Nrf2 enhances bone turnover and results in deterioration of bone structure either in sham-operated or ovariectomized mice. Our data indicate that this transcription factor is indispensable for normal bone microarchitecture and suggest that Nrf2 may be a pharmacological target to maintain bone integrity in pathological situations.
